# Identification of the *Streptococcus pyogenes* surface antigens recognised by pooled human immunoglobulin

**DOI:** 10.1038/srep15825

**Published:** 2015-10-28

**Authors:** Mark Reglinski, Magdalena Gierula, Nicola N. Lynskey, Robert J. Edwards, Shiranee Sriskandan

**Affiliations:** 1 Faculty of Medicine, Imperial College London, Hammersmith Campus, Du Cane Road, London, W12 0NN, United Kingdom

## Abstract

Immunity to common bacteria requires the generation of antibodies that promote opsonophagocytosis and neutralise toxins. Pooled human immunoglobulin is widely advocated as an adjunctive treatment for clinical *Streptococcus pyogenes* infection however, the protein targets of the reagent remain ill defined. Affinity purification of the anti-streptococcal antibodies present within pooled immunoglobulin resulted in the generation of an IgG preparation that promoted opsonophagocytic killing of *S. pyogenes in vitro* and provided passive immunity *in vivo*. Isolation of the streptococcal surface proteins recognised by pooled human immunoglobulin permitted identification and ranking of 94 protein antigens, ten of which were reproducibly identified across four contemporary invasive *S. pyogenes* serotypes (M1, M3, M12 and M89). The data provide novel insight into the action of pooled human immunoglobulin during invasive *S. pyogenes* infection, and demonstrate a potential route to enhance the efficacy of antibody based therapies.

Over the past 30 years there has been a steady rise in the global rate of severe *Streptococcus pyogenes* infection, which now exceeds 3 cases/100,000 in some regions^1^,^2^. The attendant morbidity and mortality of invasive *S. pyogenes* diseases such as necrotizing fasciitis are considerable, with a case fatality rate exceeding 40% in patients who develop streptococcal toxic shock syndrome^2^. Although the basis for population immunity to *S. pyogenes* is poorly understood, recent studies have indicated that adjunctive intravenous immunoglobulin therapy (IVIG) may confer a survival benefit during invasive *S. pyogenes* infection^3^,^4^,^5^.

IVIG is a commercially available plasmapheresis product that is purified from the blood of over one thousand healthy donors. While originally developed as a replacement therapy for hypogammaglobulinemia, the presence of specific antibodies to many human pathogens makes pooled immunoglobulin an effective prophylactic treatment for several infective conditions including hepatitis A, measles and rubella. Recent attention has focused on the protective activity of IVIG against a number of Gram positive pathogens, most notably *S. pyogenes*^3^,^4^,^5^,^6^,^7^.

Experimentally, IVIG has been shown to reduce systemic inflammation associated with streptococcal superantigen production and to promote opsonophagocytic clearance of *S. pyogenes* both *in vitro*, and in a humanised mouse model^8^,^9^. Clinically, one small trial and two population-based studies have reported benefits of IVIG administration during severe *S. pyogenes* infection and, although controversial, adjunct IVIG therapy is advocated by many physicians^3^,^4^,^5^. While some attention has focused on the varying ability of IVIG to neutralise streptococcal superantigens^10^, little is known about the *S. pyogenes* surface antigens recognised by IVIG and the mechanisms by which the reagent promotes bacterial clearance remain ill defined. One study has demonstrated the presence of anti-M1 protein antibodies within commercial preparations suggesting that IVIG may contain other antibodies that target major *S. pyogenes* surface proteins^9^. We sought to further characterise the *S. pyogenes* surface proteins recognised by IVIG and assess their protective efficacy using standard models of *S. pyogenes* infection.

## Results

### Purification of anti-streptococcal IgG from pooled immunoglobulin

**Figure 1:**
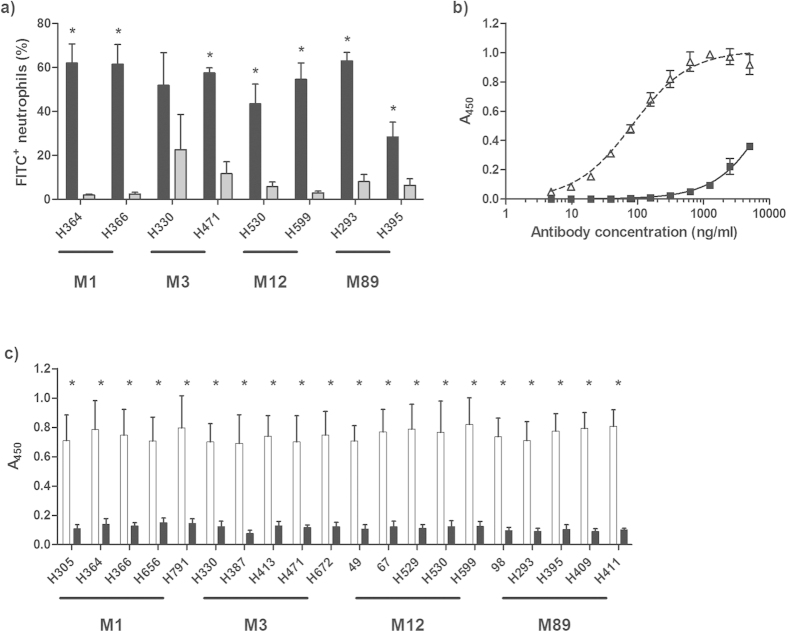
Preparation and properties of anti-streptococcal IgG (E-IVIG). (**a**) IVIG promotes neutrophil uptake of *S. pyogenes*. FITC labelled *S. pyogenes* cells were treated with 5 mg/ml of IVIG (black bars) or PBS (grey bars) and incubated with freshly isolated human neutrophils. Results from three independent experiments are expressed as percentage of FITC^+^ neutrophils (mean ± SD) after a 30 min co-incubation. n = 3, two-tailed t-test: p = 0.0003 (H364, H366 and H599); p = 0.0002; (H471); p = 0.0021 (H530); p < 0.0001 (H293); p = 0.0069 (H395). (**b**) Concentration-dependent binding of M1 *S. pyogenes* cell wall extract by E-IVIG (white triangles) and IVIG (black squares). (**c**) Serotype-independent binding of *S. pyogenes* cell wall extracts by E-IVIG (white bars) IVIG (black bars), at a fixed concentration (2500 ng/ml). Results from three independent experiments are expressed as triplicate A_450_ readings minus the background absorbance (mean ± SD). n = 3, two-tailed t-test, p < 0.0001 in all instances.

The ability of IVIG to promote phagocytic uptake of *S. pyogenes* was first confirmed using a purified human neutrophil opsonophagocytosis assay. *S. pyogenes* strains were selected to represent four of the most common contemporary serotypes associated with invasive *S. pyogenes* infection in Europe and North America (M1, M3, M12 and M89, Supplementary Table 1)^1^,^2^. At a fixed concentration of 5 mg/ml, IVIG was shown to promote neutrophil uptake of two representative strains from each serotype, all of which were isolated from invasive disease manifestations or toxic shock cases (Fig. 1A). Although the M89 strain H395 is hyperencapsulated, neutrophil uptake was still promoted by IVIG, albeit to a lesser degree than the other isolates examined. Interestingly, the baseline uptake of the M3 strain H330 was markedly higher than the other strains selected for study, reducing the apparent effect of IVIG despite comparable levels of overall uptake occurring in the presence of the reagent. This confirms that the anti-streptococcal antibodies present within pooled immunoglobulin are capable of opsonising a range of *S. pyogenes* serotypes *in vitro*.

Having established the presence of opsonic anti-streptococcal antibodies within pooled immunoglobulin, serotype M1 cell wall extract was covalently conjugated to cyanogen bromide activated agarose, and used to affinity purify the anti-streptococcal IgG fraction from commercially available IVIG. The reactivity of the resulting “enhanced” (E)-IVIG was then assessed by ELISA. The apparent affinity of E-IVIG for M1 *S. pyogenes* cell wall extract was shown to be much higher than that of the starting IVIG preparation (Fig. 1B). Furthermore, clear differences were observed in the binding of E-IVIG or IVIG to cell wall extracts from each of the 20 isolates selected for study, suggesting that the apparent increase in affinity towards *S. pyogenes* was serotype independent (Fig. 1C).

### Purification of *S. pyogenes* surface antigens by E-IVIG immunoprecipitation

In order to identify the streptococcal surface proteins targeted by IVIG, E-IVIG was covalently conjugated to cyanogen bromide activated agarose and used to purify the IVIG-reactive antigens from *S. pyogenes* cell wall extracts by immunoprecipitation. The resulting immunoprecipitates were visualised by immunoblot analysis; which confirmed the presence of a multitude of IVIG-reactive proteins within the purified preparations, none of which were present when a goat isotype control IgG column was used (Supplementary Figure 1). The immunoprecipitates were separated by SDS-PAGE, and the identity of the purified antigens was determined by proteomic analysis using a publically available proteomic database derived from two M1 *S. pyogenes* genomes (SF370 and MGAS5005).

A total of 94 E-IVIG reactive antigens were recovered from the immunoprecipitates which included several major *S. pyogenes* vaccine candidates previously described in the literature (Table 1 and Supplementary Table 2). As in previous surface proteome studies, a number of secreted and classically cytoplasmic proteins were clearly present within the starting cell wall extracts^11^,^12^. Remarkably many such proteins were purified by E-IVIG immunoprecipitation, suggesting that these molecules are capable of generating a strong humoral immune response during *S. pyogenes* infection. Of the 94 proteins identified, only ten were recovered from all 20 isolates, suggesting that these antigens are reproducibly expressed by all four invasive serotypes selected for study, and share a high degree of sequence identity between serotypes. These conserved antigens included virulence factors (C5a peptidase^13^, and SpyAD^14^), lipoproteins (maltose/maltodextrin-binding protein, oligopeptide-binding protein and nucleoside-binding protein), other cell wall-attached, LPXTG motif-containing molecules (putative pullulanase) and a single, abundant cytoplasmic protein (Chaperone protein DnaK) previously shown to stimulate *in vivo* antibody production^15^. Two of the proteins had no attributed function despite their clear degree of conservation and propensity to generate a strong humoral immune response during *S. pyogenes* infection (hypothetical membrane associated protein and cell surface protein).

To ensure that recognition of these antigens was not limited to a single source of IVIG, E-IVIG was derived from two further commercial preparations (Intratect and Privigen) and used to immunoprecipitate reactive antigens from two representative strains of each of the four *S. pyogenes* serotypes. Proteomic analysis identified the same panel of highly conserved antigens within the resulting immunoprecipitates, further implicating these molecules as important targets of adjunct IVIG therapy for invasive *S. pyogenes* infection (Supplementary Table 3).

### E-IVIG promotes opsonophagocytic killing of *S. pyogenes in vitro*

**Figure 2:**
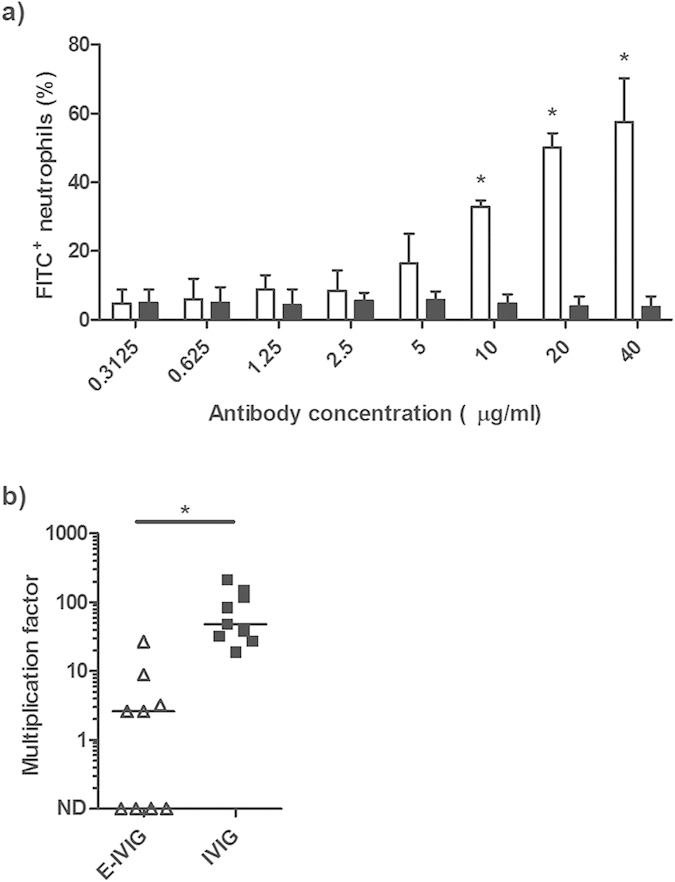
E-IVIG promotes opsonophagocytic killing of *S. pyogenes in vitro.* (**a**) FITC labelled M1 *S. pyogenes* cells (strain H364) were opsonised with doubling dilutions of E-IVIG (white bars) or IVIG (black bars) prior to incubation with freshly isolated human neutrophils. Results from three independent experiments are expressed as percentage of FITC^+^ neutrophils (mean ± SD) after a 30 min co-incubation. n = 3, two-tailed t-test: p = 0.002 (40 μg/ml); p < 0.0001 (20 μg/ml); p = 0.0001 (10 μg/ml). (**b**) Whole blood from healthy donors was supplemented with 40 μg/ml of E-IVIG (open triangles) or IVIG (closed squares) prior to inoculation with ~20 CFU of M1 *S. pyogenes* (strain H364). Results are expressed as multiplication factors (median and range). n = 9, two-tailed Mann-Whitney U: p = 0.0005. ND: Not Detected.

To determine if the higher affinity of E-IVIG for *S. pyogenes* surface antigens resulted in increased anti-streptococcal activity, the ability of E-IVIG and IVIG to promote opsonophagocytosis and killing of M1 *S. pyogenes* (strain H364) was compared. At IgG concentrations ranging from 40 μg/ml to 10 μg/ml, the percentage of neutrophils containing internalised *S. pyogenes* was significantly higher in the E-IVIG group than in the IVIG group (Fig. 2A). These results were echoed in a classical Lancefield assay of opsonophagocytic killing, where concentrations in excess of 1 mg/ml IVIG are normally required to enhance bacterial killing^8^. Supplementation of human whole blood with 40 μg/ml of E-IVIG was sufficient to increase opsonophagocytic killing by a factor of ten compared to supplementation with the same concentration of IVIG (Fig. 2B). Together these data suggest that purification of the *S. pyogenes* reactive IgG from IVIG results in a marked increase in opsonophagocytic killing through functional recognition of one or more serotype independent *S. pyogenes* surface antigens.

### Passive immunisation with E-IVIG inhibits dissemination of *S. pyogenes* from an intramuscular focus of infection

**Figure 3:**
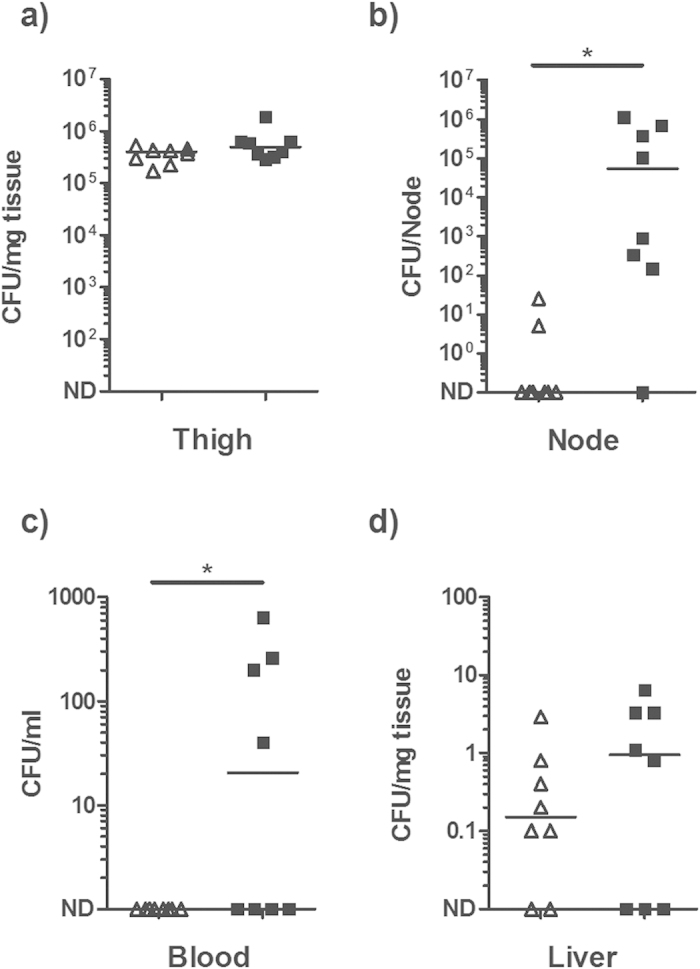
Passive immunisation with E-IVIG inhibits dissemination of *S. pyogenes* from an intramuscular focus of infection. Two groups of eight age matched C57BL/6 mice were pre-treated with 800 μg of E-IVIG (open triangles) or IVIG (closed squares) and infected intramuscularly with 5 × 10^6^ CFU of M1 *S. pyogenes* (strain H305). Solid lines indicate the median CFU recovered from (**a**) the site of original infection, (**b**) ipsilateral draining lymph node, (**c**) blood and (**d**) liver 24 h post-infection. n = 8, two-tailed Mann-Whitney U: p = 0.0037 (node); p = 0.0325 (blood). ND: Not Detected.

To assess the efficacy of E-IVIG *in vivo*, two groups of age matched C57BL/6 mice were passively immunised with E-IVIG or IVIG and challenged intramuscularly with 5 × 10^6^ CFU of M1 *S. pyogene*s (strain H305). The mice were culled 24 h post infection and the degree of bacterial dissemination was assessed by homogenisation of the organs and plating. Bacterial counts from the locally draining inguinal lymph node and blood of the E-IVIG group were significantly lower than those recovered from the IVIG group, consistent with an enhancement in bactericidal activity (Fig. 3). A similar trend was observed in the liver, although the difference was insufficient to achieve statistical significance. E-IVIG therapy had a limited effect on the bacterial burden at the site of infection, consistent with data from previous studies where treatment with antibiotics, or antibiotics plus IVIG, failed to promote bacterial clearance at the site of infection, despite promoting clearance from the blood and organs^8^. Together the data suggest that purification of the *S. pyogenes*-reactive IgG from IVIG greatly enhances the anti-streptococcal activity of the reagent both *in vitro* and *in vivo*.

## Discussion

Although IVIG is now widely advocated as an adjunctive treatment for clinical *S. pyogenes* infection, no studies have sought to characterise the repertoire of proteins recognised by the reagent. Our data demonstrate the full repertoire of proteinaceous surface antigens recognised by IVIG, and illustrate the capability of the anti-streptococcal antibody present within the reagent to promote opsonophagocytic killing of *S. pyogenes in vitro*, and provide passive protection *in vivo*, during invasive infection.

In Europe and North America, approximately 70% of all invasive *S. pyogenes* infections are caused by the ten most prominent M types^1^,^2^, four of which were represented in this study. While M1 and M3 remain the most common cause of invasive *S. pyogenes* infection, recent data suggest that has been an upsurge in invasive M89 infections over the past decade^16^,^17^. In this study E-IVIG was produced using serotype M1 cell wall extract, and a proteomic database derived from two M1 *S. pyogenes* genomes was used to identify the purified antigens. Of the 94 protein antigens isolated, only ten sequence-invariant antigens were identified from all 20 *S. pyogenes* strains selected for study. In addition to confirming that these antigens were expressed by four disparate *S. pyogenes* serotypes experimentally, the fidelity of the ten protein sequences was confirmed within all serotypes for which genome sequence data are currently available to ensure a high degree of conservation (data not shown). We considered the possibility that sequence variation and/or type-specific variation in gene content precluded proteomic identification of IVIG-reactive proteins from some isolates. However, with the exception of certain type-specific proteins such as the M protein and pilus, the core genomes of different *S. pyogenes* serotypes are largely conserved in sequence, and gene content^18^. Indeed, selective re-analysis of the purified immunoprecipitates using separate M3, M12 and M89 proteomic databases did not impact appreciably on our findings (data not shown). Therefore the ten highly conserved, invariant antigens identified herein may be of particular importance for the reported efficacy of adjunct IVIG therapy, and may warrant consideration as components of an effective anti-*S. pyogenes* vaccine.

Interestingly, despite the use of pooled M1 cell wall extract for affinity purification of E-IVIG, and an M1 database for proteomic analysis of the resulting immunoprecipitates, the M protein was identified in 14/20 strains evaluated. The apparent absence of the M protein from the M89 preparations could be wholly attributed to substantial differences between the M1 and M89 protein sequences; and reanalysis of the M89 immunoprecipitate data using an *emm*89 sequence clearly identified M protein from all five strains selected for study (data not shown). Therefore, despite considerable variation between the different *emm* sequences, a substantial amount of cross reactive anti-M protein antibody was clearly present within the affinity purified E-IVIG preparation. This finding supports the existence of cross protective epitopes within disparate M protein isoforms^19^.

Repeated exposure of the humoral immune system to a variety of common pathogens results in the accumulation of circulating antibodies with a diverse range of specificities. Affinity purification of anti-streptococcal antibodies from IVIG resulted in the generation of an antibody preparation with enhanced activity against *S. pyogenes in vivo*. While the breadth of protection may still be limited by serotype or strain specific differences in protein expression, E-IVIG may represent a more effective adjunct treatment for invasive *S. pyogenes* infection than the polyspecific IVIG that is currently employed clinically. It is possible that hyperencapsulation of *S. pyogenes* may impact negatively on the efficacy of therapeutic opsonic antibodies through masking of surface antigens, especially in strains that possess mutations in the global virulence regulator CovR/S^20^. However, IVIG was capable of promoting opsonophagocytic uptake of the hyperencapsulated *covS* mutant H395^16^, albeit at a reduced level (Fig. 1). This suggests that, while some of the epitopes targeted by IVIG may be shielded from antibody recognition by the capsule, a crucial subset remain exposed in hyperencapsulated isolates.

Although not intended to target secreted proteins, a number of extracellular toxins were purified from the cell wall extracts by E-IVIG immunoprecipitation, including streptolysin-O and the cysteine protease SpeB. Intriguingly, the repertoire of purified secreted proteins did not include any superantigens, which play a key role during invasive *S. pyogenes* infection and are strong targets of the human humoral immune response^10^,^21^. Thus further development of E-IVIG for *S. pyogenes* sepsis will have to address the possibility that the broadly acting anti-superantigen activity of IVIG may not be maintained following anti-streptococcal antibody enrichment. Furthermore, any pre-clinical evaluation of E-IVIG will require rigorous screening to ensure the purity of the antibody preparation and to rule out any unforeseen toxicity resulting from the carryover of bacterial products. Nonetheless, in light of the shortage of human immunoglobulin, it may be prudent to determine whether therapeutic antibodies against a wider range of pathogens could be sequentially isolated from a single IVIG preparation using multiple rounds of affinity purification. Such targeted reagents may prove valuable for the adjunct treatment of a wide range of common antimicrobial-resistant pathogens. Indeed while here we have applied immunoprecipitation to *S. pyogenes*, published reports and our own unpublished data suggest that the technique could equally be applied to other Gram positive bacteria such as *Staphylococcus aureus* and *Enterococcus* spp. where clearance is mediated through similar processes^6^,^7^,^22^.

As a pooled preparation of immunoglobulin from normal donor blood, IVIG is an obvious but hitherto unevaluated reagent for the systematic identification of the antigens recognised by the humoral immune response of a healthy human population. Previous studies have attempted to characterise the streptococcal antigens targeted by the human antibody response, however the results may have been influenced by the use of non-native protein preparations and/or serum of unknown protective activity, which is often employed for screening target antigens^12^,^23^,^24^,^25^. The data in this study identify for the first time a core list of highly conserved *S. pyogenes* antigens against which protective humoral immunity is directed. The findings enhance our understanding of population immunity to *S. pyogenes* as a whole, and may have implications for both passive and active vaccination.

## Materials and Methods

### Bacterial strains and growth conditions

The *S. pyogenes* isolates used are listed in Supplementary Table 1 and were routinely cultured on Columbia horse blood agar (CBA) or in Todd-Hewitt broth (THB) at 37 °C in 5% CO_2_.

### Preparation of streptococcal cell wall extracts and immunoblotting

Streptococcal cell wall extracts were isolated as previously described^26^. Briefly, overnight *S. pyogenes* cultures were diluted 1:10 in fresh THB and grown to an A_600_ of 0.4–0.8. The cells were pelleted and resuspended in 1/50th volume of 10 mM Tris-HCl (pH 8.0) containing 30% raffinose, 100 U/ml mutanolysin, 1 mg/ml lysozyme and 10% Protease Inhibitor Cocktail Set III (Calbiochem), and incubated for 3 h at 37 °C with occasional inversion. The cells were pelleted and the supernatant (cell wall extract) was dialysed into PBS overnight and concentrated by centrifugation (10,000 MWCO). For immunoblotting, proteins were separated on 7% tris-acetate (TA) gels (Invitrogen) and transferred to Hybond LFP membranes (GE Healthcare). Membranes were blocked with 5% skimmed milk prior to the addition of 4 μg/ml IVIG (Endobulin^®^, Baxter). Bound antibodies were detected using a 1:80,000 dilution of HRP-conjugated goat anti human IgG (Sigma-Aldrich) and the ECL prime detection system (GE Healthcare).

### Affinity purification of E-IVIG and immunoprecipitation of reactive streptococcal surface antigens

Pooled serotype M1 cell wall extract was dialysed into coupling buffer (0.1 M sodium bicarbonate, 0.5 M sodium chloride, pH 8.3) overnight and coupled to cyanogen bromide (CNBr) activated agarose at a concentration of ~1 mg/ml according to the manufacturer’s instructions (Sigma-Aldrich). Briefly, lyophilised CNBr-activated agarose was swollen in cold 1 mM HCl (200 ml/g dry gel), pipetted into a 5 ml column and washed sequentially with ten column volumes of distilled water and five column volumes of coupling buffer. The dialysed cell wall extract was immediately added and incubated with the swollen resin overnight at 4 °C. The resin was washed extensively with coupling buffer and incubated with two column volumes of 0.2 M glycine at RT for 2 h with gentle agitation. The resin was washed sequentially with ten column volumes of coupling buffer and ten column volumes of sodium acetate buffer (0.1 M anhydrous sodium acetate, 0.5 M sodium chloride, pH 4). This high/low pH wash cycle was repeated five times and the resulting affinity purification column was equilibrated into PBS supplemented with 0.1% sodium azide. For affinity purification, the resin was incubated with 5 ml of 5 mg/ml IVIG for 2 h at RT and washed extensively with PBS. Bound antibody was eluted into 1 ml aliquots of 1 M acetic acid and immediately neutralised using an equal volume of 3 M Tris-HCl (pH 8.8). Eluted fractions (containing the E-IVIG) were pooled, dialysed into PBS and concentrated. E-IVIG was stored in 0.1% sodium azide at 4 °C until required. For immunoprecipitation, E-IVIG or goat isotype control IgG (Abcam) was coupled to CNBr-activated agarose at a concentration of ~1 mg/ml, and incubated with 2 ml aliquots of concentrated cell wall extract. The resin was washed extensively and the bound antigens were eluted, dialysed and concentrated as outlined above. For comparative immunoprecipitation experiments, additional E-IVIG preparations were prepared from Intratect^®^ (Biotest) and Privigen^®^ (CSL Behring) IVIG.

### Analysis of E-IVIG by ELISA

To compare binding of E-IVIG and IVIG, 96 well plates were coated with 1 μg/well of cell wall extract and incubated with 2.5 mg/ml of antibody. Bound antibodies were detected using a 1: 80,000 dilution of HRP-conjugated goat anti-human IgG and 50 μl/well tetramethylbenzidine. The detection reaction was stopped through the addition of 50 μl of 1 M H_2_SO_4_ and the absorbance at 450 nm read using a μQuant universal microplate spectrophotometer (Bio-tek instruments).

### Opsonophagocytic uptake and killing assays

Human neutrophil opsonophagocytosis assays were performed using normal donor neutrophils obtained with informed consent from a subcollection of the Imperial College Tissue Bank and FITC-labelled *S. pyogenes* as previously described^27^. Briefly, bacterial cells from a 5 ml overnight culture were pelleted, washed and incubated in 500 μl of 20 μg/ml FITC in 0.1 M sodium carbonate buffer (pH 9) for 30 min at 37 °C. The cells were washed twice and resuspended to an A_600_ of 0.35 in PBS and incubated with an appropriate concentration of E-IVIG, IVIG or PBS at 37 °C for 30 min with agitation. Neutrophils were purified from heparinised whole blood that was combined 1:1 with 3% dextran (MW > 100,000) in 0.9% saline and allowed to separate on ice for 30 min. The leukocytes present in the supernatant layer were pelleted and resuspended in one volume of 0.9% saline. Neutrophils were then purified by Ficoll-layering as described previously^28^. Opsonophagocytosis assays were performed in 1 ml reaction volumes containing 100 μl of resuspended bacteria and 1 × 10^6^ neutrophils. After a 30 min co-incubation, the *S. pyogenes*/neutrophil suspension was combined 1:1 with 0.02% EDTA in PBS and, immediately prior to analysis, the extracellular fluorescence was quenched through addition of one volume of trypan blue. Results are expressed as the percentage of neutrophils containing FITC^+^ bacteria as determined by flow cytometry. The strains selected were all isolated from invasive disease or toxic shock cases (Supplementary table 1). Whole blood opsonophagocytic killing (Lancefield) assays were performed under standard conditions using whole blood from three healthy human donors supplemented with 40 μg/ml of IVIG or E-IVIG and approximately 20 CFU of M1 *S. pyogenes* (strain H364) using a standard method^8^,^29^,^30^. Results from nine independent reactions were expressed as the bacterial multiplication factor following 3 h incubation at 37 °C with gentle agitation.

### Proteomic analysis

Immunoprecipitated antigens were separated on 7% TA gels and subjected to in gel trypsin digest and subsequent liquid chromatography-tandem mass spectrometry as previously described^31^. Proteins were identified using a publically available proteome for serotype M1 *S. pyogenes* (Uniprot, CP000017)^32^,^33^; and correlation of the MS/MS peptide data with the *in silico* trypsin fragmentation patterns predicted using BioWorks 3.3 and the SEQUEST algorithm (Thermo Scientific). Peptides generating ions of 200–2000 amu, with a cross correlation score >1.5, 2.0, or 2.5 (where z = 1, 2 or 3 respectively) and a p < 0.01 were mapped onto the *S. pyogenes* proteome. Proteins that contained two or more of the identified peptides were selected for further analysis. The protein probability for each selected protein was calculated using the SEQUEST algorithm and reported as a range of up to 20 values derived independently from analysis of proteins from each of the strains examined.

### Passive immunisation

5–6 week old female C57BL/6 mice were passively immunised with 800 μg of E-IVIG or IVIG in 100 μl of PBS by intraperitoneal injection. 16 h after IgG administration, the mice were challenged intramuscularly with 5 × 10^6^ CFU of M1 *S. pyogenes* (Strain H305 which has been used previously in IVIG protection studies^8^). After 24 h, the mice were culled, the blood and organs were removed and bacterial loads at the infection site and the degree of dissemination were assessed by plating serial dilutions of each organ homogenate onto CBA. All animal procedures were approved by the local ethical review process at Imperial College London and conducted in accordance with the relevant, UK Home Office approved, project license.

### Statistical analysis

Data are expressed as mean and standard deviation (bar charts and line graphs) or median and range (scatter plots) and analysed using the two-tailed student’s t-test or two-tailed Mann-Whitney U where appropriate. Statistical significance was accepted where *p < 0.05. Curve fits were calculated by non-linear regression (GraphPad Prism).

## Additional Information

**How to cite this article**: Reglinski, M. *et al.* Identification of the *Streptococcus pyogenes* surface antigens recognised by pooled human immunoglobulin. *Sci. Rep.* **5**, 15825; doi: 10.1038/srep15825 (2015).

## Supplementary Material

Supplementary Information

## Figures and Tables

**Table 1:** The *S. pyogenes* antigens purified by E-IVIG immunoprecipitation.

Uniprot identifier	Protein product	SEQUEST P-values (range)	M1	M3	M12	M89	Total	
C5AP_STRP1	C5a peptidase	1 × 10^−30^ −7 × 10^−10^	5/5	5/5	5/5	5/5	20/20	100%
DNAK_STRP1	Chaperone protein DnaK	8 × 10^−15^ −8 × 10^−8^	5/5	5/5	5/5	5/5	20/20	
Q48Y96_STRP1	Maltose/maltodextrin-binding protein	2 × 10^−16^ −3 × 10^−10^	5/5	5/5	5/5	5/5	20/20	
Q490V0_STRP1	Oligopeptide-binding protein	1 × 10^−30^ −2 × 10^−10^	5/5	5/5	5/5	5/5	20/20	
Q491G2_STRP1	Nucleoside-binding protein	1 × 10^−30^ −1 × 10^−15^	5/5	5/5	5/5	5/5	20/20	
Q99XX8_STRP1	Putative pullulanase	2 × 10^−14^ −6 × 10^−8^	5/5	5/5	5/5	5/5	20/20	
Q99ZH4_STRP1	Nucleoside-binding protein	1 × 10^−30^ −7 × 10^−11^	5/5	5/5	5/5	5/5	20/20	
Q99ZW9_STRP1	Hypothetical membrane associated protein	1 × 10^−30^ −2 × 10^−13^	5/5	5/5	5/5	5/5	20/20	
Q9A0C0_STRP1	Cell surface protein	7 × 10^−14^ −3 × 10^−7^	5/5	5/5	5/5	5/5	20/20	
Q9A1H3_STRP1	SpyAD	2 × 10^−15^ −4 × 10^−11^	5/5	5/5	5/5	5/5	20/20	
EFTS_STRP1	Elongation factor Ts	3 × 10^−15^ −3 × 10^−8^	5/5	5/5	4/5	5/5	19/20	70-95%
MTSA_STRP1	Metal ABC transporter substrate-binding lipoprotein	1 × 10^−30^ −9 × 10^−10^	5/5	5/5	4/5	5/5	19/20	
PGK_STRP1	Phosphoglycerate kinase	6 × 10^−14^ −5 × 10^−9^	5/5	3/5	5/5	5/5	18/20	
Q99YL6_STRP1	Putative uncharacterised protein	2 × 10^−9^ −2 × 10^−6^	4/5	4/5	5/5	5/5	18/20	
Q9A0C5_STRP1	Putative uncharacterised protein	1 × 10^−10^ −6 × 10^−5^	5/5	3/5	5/5	5/5	18/20	
TACY_STRP1	Streptolysin O	1 × 10^−30^ −5 × 10^−7^	5/5	5/5	5/5	3/5	18/20	
PEPDB_STRP1	Probable dipeptidase B	1 × 10^−30^ −2 × 10^−7^	4/5	3/5	5/5	5/5	17/20	
Q7DAN2_STRP1	Nicotine adenine dinucleotide glycohydrolase	8 × 10^−15^ −3 × 10^−11^	5/5	5/5	5/5	2/5	17/20	
Q9A1G0_STRP1	Penicillin-binding protein	9 × 10^−12^ −1 × 10^−4^	2/5	5/5	5/5	5/5	17/20	
Q490K8_STRP1	SpyCEP	3 × 10^−15^ −1 × 10^−5^	5/5	5/5	2/5	4/5	16/20	
Q99XU2_STRP1	Periplasmic component of efflux system	3 × 10^−13^ −9 × 10^−9^	5/5	3/5	3/5	5/5	16/20	
Q48XJ9_STRP1	Sugar-binding protein	3 × 10^−15^ −2 × 10^−5^	5/5	3/5	1/5	5/5	14/20	
Q99XV0_STRP1	M protein	5 × 10^−13^ −5 × 10^−6^	5/5	5/5	4/5	0/5	14/20	

Numbers represent the proportion of strains from which each target was identified. Targets identified from ≥70% of strains are displayed.
