# Supplementary material for: Identification of the Streptococcus pyogenes surface antigens recognised by pooled human immunoglobulin
Source: Sci Rep. 2015 Oct 28;5:15825. doi: 10.1038/srep15825 (PMC4623672; doi:10.1038/srep15825)
Supplement: Supplementary Information [file srep15825-s1.pdf]

Title: Identification of the *Streptococcus pyogenes* surface antigens recognised by pooled human immunoglobulin

Authors: Mark Reglinski, Magdalena Gierula, Nicola N Lynskey, Robert J Edwards and *Shiranee Sriskandan

Affiliations: Faculty of Medicine, Imperial College London, Hammersmith Campus, Du Cane Road, London, W12 0NN, United Kingdom

***Corresponding author:** Shiranee Sriskandan

Address: Section of Infectious Diseases, Imperial College London, Hammersmith Campus, London, W12 0NN

Email: s.sriskandan@imperial.ac.uk

Supporting information

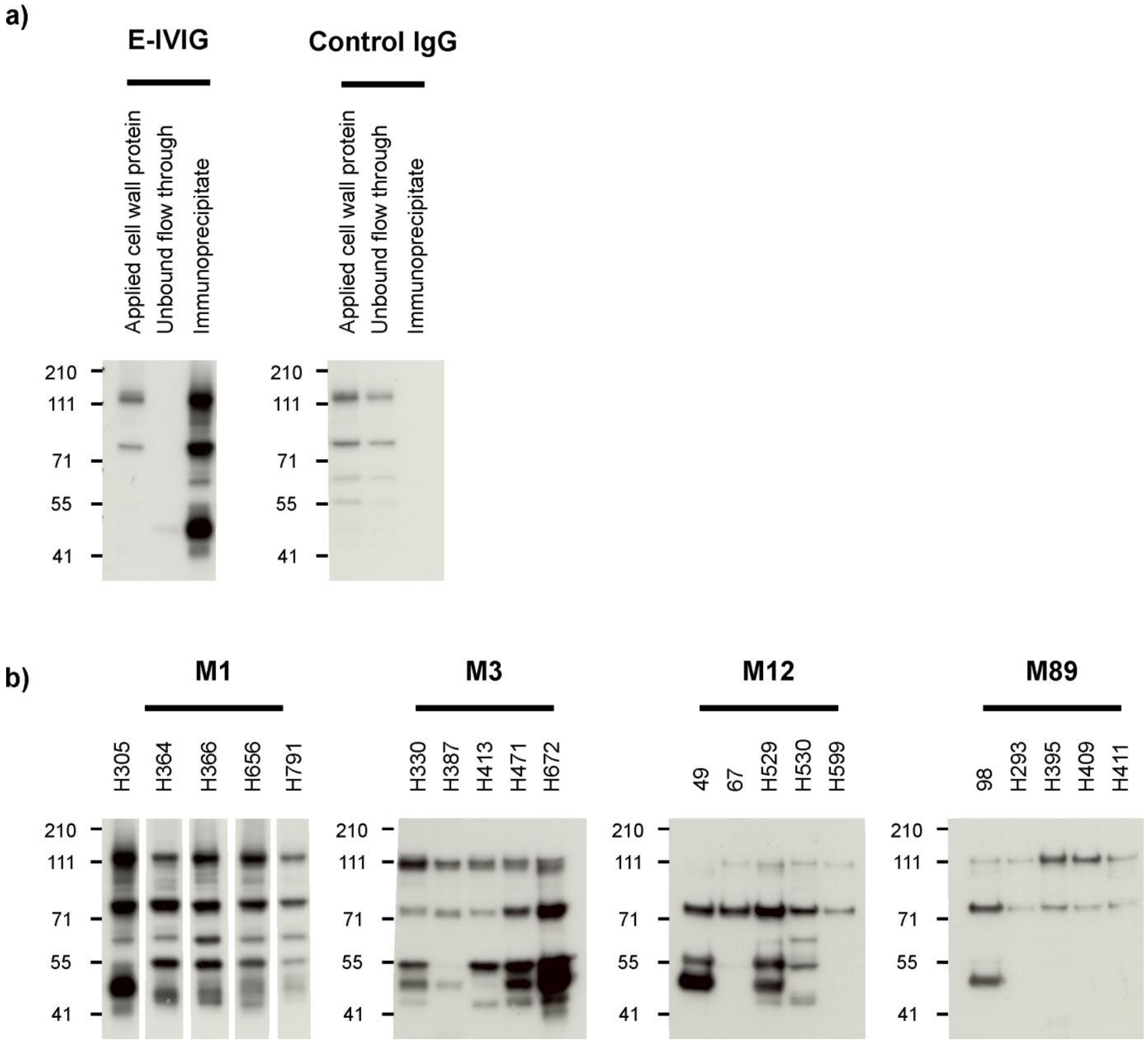

Supplementary Figure 1: Purification of *S. pyogenes* surface antigens by E-IVIG immunoprecipitation. A) In preliminary experiments antigenic proteins were purified from serotype M1 cell wall extract (strain H305) by immunoprecipitation using E-IVIG or polyclonal goat (control) IgG. Aliquots of the applied cell wall extract, the column flow through and the resulting immunoprecipitate were separated on 7% tris-acetate gels and subjected to immunoblot analysis using IVIG as the primary antibody. B) The immunoprecipitates recovered from the 20 strains selected for study were separated on 7% TA gels and subjected to immunoblot analysis using IVIG as the primary antibody. Molecular mass markers are given in kilodaltons.

Supplementary Table 1: *S. pyogenes* isolates used in this study

M type/Isolate	Source	Disease manifestation
**M1**		
H305	Throat	Scarlet fever
H364*	Lung	Pneumonia/septic shock
H366*	Blood	Pneumonia/septic shock
H656	Blood	Bacteremia
H791	Blood	Cellulitis
**M3**		
H330*	Blood	Puerperal sepsis
H387	Throat	Septic shock
H413	-	Septic shock
H471*	Ear	Septic shock
H672	Throat	Tonsillitis
**M12**		
49	Throat	Tonsillitis
67	Throat	Tonsillitis
H529	Blood	Septic shock
H530*	Blood	Septic shock
H599*	Blood	Bacteremia
**M89**		
98†	Throat	Tonsillitis
H293*	Muscle	Necrotizing fasciitis/septic shock
H395‡	Blood	Pneumonia
H409	Invasive	Septic shock
H411	Blood	Bacteremia

***Isolates used in opsonophagocytic uptake assays**
†Genetically acapsular [16]
‡Hyperencapsulated *covS* mutant [16]

Supplementary Table 2: The *S. pyogenes* antigens purified by E-IVIG immunoprecipitation. Numbers represent the proportion of strains from which targets were identified.

Uniprot identifier	Gene	Protein product	SEQUEST P-values (range)	M1	M3	M12	M89	Total	
C5AP_STRP1	*scpA, SPy_2010, M5005_Spy1715*	C5a peptidase	$1x10^{-30} - 7x10^{-10}$	5/5	5/5	5/5	5/5	20/20	
DNAK_STRP1	*dnaK, SPy_1760, M5005_Spy1498*	Chaperone protein DnaK	$8x10^{-15} - 8x10^{-8}$	5/5	5/5	5/5	5/5	20/20	
Q48Y96_STRP1	*malE, M5005_Spy1058, SPy_1294*	Maltose/maltodextrin-binding protein	$2x10^{-16} - 3x10^{-10}$	5/5	5/5	5/5	5/5	20/20	
Q490V0_STRP1	*oppA, M5005_Spy0249*	Oligopeptide-binding protein	$1x10^{-30} - 2x10^{-10}$	5/5	5/5	5/5	5/5	20/20	
Q491G2_STRP1	*M5005_Spy0137*	Nucleoside-binding protein	$1x10^{-30} - 1x10^{-15}$	5/5	5/5	5/5	5/5	20/20	
Q99XX8_STRP1	*pulA, SPy_1972*	Putative pullulanase	$2x10^{-14} - 6x10^{-8}$	5/5	5/5	5/5	5/5	20/20	100%
Q99ZH4_STRP1	*M5005_Spy0942, SPy_1228*	Nucleoside-binding protein	$1x10^{-30} - 7x10^{-11}$	5/5	5/5	5/5	5/5	20/20	
Q99ZW9_STRP1	*M5005_Spy0762, SPy_1037*	Hypothetical membrane associated protein	$1x10^{-30} - 2x10^{-13}$	5/5	5/5	5/5	5/5	20/20	
Q9A0C0_STRP1	*M5005_Spy0651, SPy_0843*	Cell surface protein	$7x10^{-14} - 3x10^{-7}$	5/5	5/5	5/5	5/5	20/20	
Q9A1H3_STRP1	*prgA, M5005_Spy0229, SPy_0269*	SpyAD	$2x10^{-15} - 4x10^{-11}$	5/5	5/5	5/5	5/5	20/20	
EFTS_STRP1	*tsf, SPy_2093, M5005_Spy1781*	Elongation factor Ts	$3x10^{-15} - 3x10^{-8}$	5/5	5/5	4/5	5/5	19/20	
MTSA_STRP1	*mtsA, SPy_0453, M5005_Spy0368*	Metal ABC transporter substrate-binding lipoprotein	$1x10^{-30} - 9x10^{-10}$	5/5	5/5	4/5	5/5	19/20	
PGK_STRP1	*pgk, SPy_1881, M5005_Spy1599*	Phosphoglycerate kinase	$6x10^{-14} - 5x10^{-9}$	5/5	3/5	5/5	5/5	18/20	
Q99YL6_STRP1	*SPy_1643*	Putative uncharacterized protein	$2x10^{-9} - 2x10^{-6}$	4/5	4/5	5/5	5/5	18/20	
Q9A0C5_STRP1	*SPy_0836*	Putative uncharacterized protein	$1x10^{-10} - 6x10^{-5}$	5/5	3/5	5/5	5/5	18/20	
TACY_STRP1	*slo, SPy_0167, M5005_Spy0141*	Streptolysin O	$1x10^{-30} - 5x10^{-7}$	5/5	5/5	5/5	3/5	18/20	
PEPDB_STRP1	*pepDB, SPy_2066, M5005_Spy1758*	Probable dipeptidase B	$1x10^{-30} - 2x10^{-7}$	4/5	3/5	5/5	5/5	17/20	70-95%
Q7DAN2_STRP1	*nga, SPy_0165*	Nicotine adenine dinucleotide glycohydrolase	$8x10^{-15} - 3x10^{-11}$	5/5	5/5	5/5	2/5	17/20	
Q9A1G0_STRP1	*dacA, dacA2, M5005_Spy0248, SPy_0292*	Penicillin-binding protein (D-alanyl-D-alanine carboxypeptidase)	$9x10^{-12} - 1x10^{-4}$	2/5	5/5	5/5	5/5	17/20	
Q490K8_STRP1	*M5005_Spy0341*	SpyCEP	$3x10^{-15} - 1x10^{-5}$	5/5	5/5	2/5	4/5	16/20	
Q99XU2_STRP1	*M5005_Spy1728, SPy_2032*	Periplasmic component of efflux system	$3x10^{-13} - 9x10^{-9}$	5/5	3/5	3/5	5/5	16/20	
Q48XJ9_STRP1	*M5005_Spy1308, SPy_1592*	Sugar-binding protein	$3x10^{-15} - 2x10^{-5}$	5/5	3/5	1/5	5/5	14/20	
Q99XV0_STRP1	*emm1, SPy_2018*	M protein type 1	$5x10^{-13} - 5x10^{-6}$	5/5	5/5	4/5	0/5	14/20	
EFG_STRP1	*fus, SPy_0273, M5005_Spy0232*	Elongation factor G	$6x10^{-16} - 8x10^{-5}$	5/5	2/5	2/5	4/5	13/20	
Q9A147_STRP1	*M5005_Spy0384, SPy_0469*	Putative 42 kDa protein	$7x10^{-10} - 7x10^{-4}$	5/5	1/5	4/5	3/5	13/20	
PURA_STRP1	*purA, SPy_0160, M5005_Spy0136*	Adenylosuccinate synthetase	$2x10^{-13} - 3x10^{-5}$	5/5	2/5	2/5	3/5	12/20	
Q48Y78_STRP1	*glnH, M5005_Spy1076*	Transporter	$1x10^{-30} - 4x10^{-6}$	3/5	2/5	5/5	2/5	12/20	
Q99ZT1_STRP1	*M5005_Spy0818, SPy_1094*	Polysaccharide deacetylase	$8x10^{-10} - 8x10^{-7}$	3/5	0/5	5/5	4/5	12/20	
PILIN_STRP1	*SPy_0128, M5005_Spy0109*	Pilin	$1x10^{-15} - 1x10^{-13}$	5/5	5/5	1/5	0/5	11/20	
Q48ZH8_STRP1	*M5005_Spy0622*	Phosphoglycerol transferase	$3x10^{-13} - 3x10^{-5}$	5/5	1/5	1/5	4/5	11/20	50-65%
Q491J2_STRP1	*M5005_Spy0107*	Fibronectin-binding protein	$1x10^{-30} - 6x10^{-7}$	5/5	5/5	1/5	0/5	11/20	
Q99YQ4_STRP1	*SPy_1591*	Putative uncharacterized protein	$2x10^{-11} - 2x10^{-4}$	4/5	1/5	1/5	5/5	11/20	
Q99Z76_STRP1	*inlA, M5005_Spy1109, SPy_1361*	Internalin protein	$1x10^{-11} - 1x10^{-6}$	4/5	4/5	2/5	1/5	11/20	
SPEB_STRP1	*speB, SPy_2039, M5005_Spy1735*	Streptopain	$4x10^{-15} - 1x10^{-6}$	5/5	1/5	1/5	4/5	11/20	
Q99ZX4_STRP1	*hylA, M5005_Spy0757, SPy_1032*	Extracellular hyaluronate lyase	$2x10^{-15} - 7x10^{-4}$	5/5	0/5	3/5	2/5	10/20	
Q9A0J7_STRP1	*M5005_Spy0571, SPy_0747*	Endonuclease/exonuclease/phosphatase family protein	$6x10^{-14} - 1x10^{-6}$	5/5	1/5	2/5	2/5	10/20	

Supplementary Table 2 continued:

Uniprot identifier	Gene	Protein product	SEQUEST P-values (range)	M1	M3	M12	M89	Total	
ENO_STRP1	*eno, SPy_0731, M5005_Spy0556*	Enolase	$1x10^{-30}$ - $5x10^{-9}$	4/5	1/5	2/5	2/5	9/20	
G3P_STRP1	*gap, gapA, plr, SPy_0274, M5005_Spy0233*	Glyceraldehyde-3-phosphate dehydrogenase	$1x10^{-30}$ - $1x10^{-6}$	5/5	1/5	1/5	2/5	9/20	
Q491G0_STRP1	*nga, M5005_Spy0139*	NAD glycohydrolase	$6x10^{-13}$ - $2x10^{-7}$	4/5	3/5	2/5	0/5	9/20	
Q99ZB1_STRP1	*malX, M5005_Spy1067, SPy_1306*	Maltose/maltodextrin-binding protein	$6x10^{-15}$ - $5x10^{-9}$	5/5	0/5	0/5	4/5	9/20	
CH60_STRP1	*groL, groEL, SPy_2070, M5005_Spy1761*	60 kDa chaperonin	$6x10^{-13}$ - $1x10^{-7}$	5/5	1/5	2/5	0/5	8/20	
Q9A1U2_STRP1	*pbp1b, M5005_Spy0082, SPy_0097*	Multimodular transpeptidase-transglycosylase PBP 1B	$2x10^{-9}$ - $1x10^{-5}$	5/5	0/5	1/5	2/5	8/20	
Q9A209_STRP1	*dnaN, M5005_Spy0002, SPy_0003*	DNA polymerase III subunit beta	$4x10^{-9}$ - $3x10^{-7}$	5/5	0/5	3/5	0/5	8/20	
Q99XV4_STRP1	*M5005_Spy1710, SPy_2006*	Streptococcal histidine triad protein	$7x10^{-13}$ - $3x10^{-8}$	5/5	0/5	1/5	1/5	7/20	
Q9A1S0_STRP1	*M5005_Spy0111, SPy_0130*	Putative uncharacterized protein	$6x10^{-13}$ - $8x10^{-5}$	5/5	2/5	0/5	0/5	7/20	
STRP_STRP1	*ska, SPy_1979, M5005_Spy1684*	Streptokinase A	$1x10^{-30}$ - $1x10^{-4}$	4/5	2/5	1/5	0/5	7/20	
Q99Y99_STRP1	*isp2, M5005_Spy1531, SPy_1801*	Immunogenic secreted protein	$3x10^{-10}$ - $1x10^{-5}$	4/5	2/5	0/5	0/5	6/20	25-45%
Q99ZD1_STRP1	*pyk, M5005_Spy0988, SPy_1282*	Pyruvate kinase	$2x10^{-11}$ - $1x10^{-5}$	4/5	0/5	1/5	1/5	6/20	
Q9A180_STRP1	*prtS, SPy_0416*	Putative cell envelope proteinase	$7x10^{-5}$ - $8x10^{-4}$	1/5	3/5	0/5	2/5	6/20	
Q9A1I9_STRP1	*M5005_Spy0213, SPy_0252*	N-acetylneuraminate-binding protein	$5x10^{-12}$ - $9x10^{-10}$	5/5	0/5	0/5	1/5	6/20	
Q9A1Z8_STRP1	*sibA, M5005_Spy0017, SPy_0019*	Putative secreted protein	$2x10^{-13}$ - $7x10^{-8}$	5/5	0/5	1/5	0/5	6/20	
ADCA_STRP1	*adcA, SPy_0714, M5005_Spy0543*	Zinc-binding protein AdcA	$7x10^{-12}$ - $8x10^{-7}$	4/5	0/5	1/5	0/5	5/20	
K6PF_STRP1	*pfkA, pfk, SPy_1283, M5005_Spy0989*	6-phosphofructokinase	$1x10^{-30}$ - $2x10^{-4}$	4/5	0/5	1/5	0/5	5/20	
Q48WW7_STRP1	*endoS, M5005_Spy1540*	Endo-beta-N-acetylglucosaminidase F2	$1x10^{-15}$ - $6x10^{-7}$	1/5	0/5	1/5	3/5	5/20	
Q99XQ6_STRP1	*pepO, SPy_2095*	Putative endopeptidase O	$2x10^{-13}$ - $6x10^{-5}$	3/5	2/5	0/5	0/5	5/20	
Q99YF1_STRP1	*lytR, M5005_Spy1474, SPy_1733*	Putative transcription regulator	$6x10^{-14}$ - $3x10^{-9}$	5/5	0/5	0/5	0/5	5/20	
TIG_STRP1	*tig, ropA, SPy_1896, M5005_Spy1612*	Trigger factor	$7x10^{-8}$ - $6x10^{-5}$	5/5	0/5	0/5	0/5	5/20	

Supplementary Table 2 continued:

Uniprot identifier	Gene	Protein product	SEQUEST P-values (range)	M1	M3	M12	M89	Total	
ALF_STRP1	*fba, SPy_1889, M5005_Spy1607*	Fructose-bisphosphate aldolase	$3x10^{-13}$ - $2x10^{-5}$	0/5	3/5	0/5	1/5	4/20	
IMDH_STRP1	*guaB, SPy_2206, M5005_Spy1857*	Inosine-5'-monophosphate dehydrogenase	$2x10^{-12}$ - $3x10^{-7}$	4/5	0/5	0/5	0/5	4/20	
Q48YY7_STRP1	*dacA1, M5005_Spy0817*	D-alanyl-D-alanine carboxypeptidase	$4x10^{-12}$ - $4x10^{-8}$	4/5	0/5	0/5	0/5	4/20	
Q99XZ6_STRP1	*M5005_Spy1659, SPy_1945*	Putative uncharacterized protein	$3x10^{-11}$ - $4x10^{-8}$	1/5	2/5	1/5	0/5	4/20	
Q99YN0_STRP1	*M5005_Spy1335, SPy_1625*	Serine/threonine protein kinase	$5x10^{-12}$ - $1x10^{-4}$	4/5	0/5	0/5	0/5	4/20	
Q9A156_STRP1	*SPy_0457*	Putative cyclophilin-type protein	$7x10^{-7}$ - $2x10^{-5}$	0/5	0/5	4/5	0/5	4/20	
EFTU_STRP1	*tuf, SPy_0611, M5005_Spy0508*	Elongation factor Tu	$1x10^{-14}$ - $3x10^{-11}$	3/5	0/5	0/5	0/5	3/20	
OTCC_STRP1	*arcB, SPy_1544, M5005_Spy1273*	Ornithine carbamoyltransferase, catabolic	$2x10^{-12}$ - $8x10^{-10}$	2/5	0/5	0/5	1/5	3/20	
PRSA1_STRP1	*prsA1, SPy_1390, M5005_Spy1133*	Foldase protein prsA 1	$2x10^{-12}$ - $3x10^{-11}$	3/5	0/5	0/5	0/5	3/20	
Q48ZN9_STRP1	*epf, M5005_Spy0561*	Putative extracellular matrix binding protein	$1x10^{-7}$ - $2x10^{-5}$	2/5	1/5	0/5	0/5	3/20	
Q99ZB3_STRP1	*amyA, M5005_Spy1065, SPy_1302*	Alpha-amylase	$3x10^{-15}$ - $8x10^{-5}$	3/5	0/5	0/5	0/5	3/20	
Q9A0A2_STRP1	*M5005_Spy0678, SPy_0872*	5'-nucleotidase	$5x10^{-9}$ - $3x10^{-5}$	2/5	1/5	0/5	0/5	3/20	
Q9A1H5_STRP1	*cbf, M5005_Spy0227, SPy_0267*	CMP-binding factor	$7x10^{-8}$ - $3x10^{-7}$	3/5	0/5	0/5	0/5	3/20	
LDH_STRP1	*ldh, SPy_1151, M5005_Spy0873*	L-lactate dehydrogenase	$5x10^{-9}$ - $6x10^{-5}$	2/5	0/5	0/5	0/5	2/20	
Q48WD4_STRP1	*isp, M5005_Spy1723*	Immunogenic secreted protein	$6x10^{-6}$ - $7x10^{-6}$	2/5	0/5	0/5	0/5	2/20	
Q48YT2_STRP1	*nox, M5005_Spy0872*	NADH oxidase H2O-forming	$1x10^{-8}$ - $6x10^{-8}$	2/5	0/5	0/5	0/5	2/20	
Q48Z64_STRP1	*cpsFQ, M5005_Spy0736*	dTDP-glucose 4,6-dehydratase	$2x10^{-7}$ - $4x10^{-4}$	2/5	0/5	0/5	0/5	2/20	
Q490V2_STRP1	*M5005_Spy0247*	Ribonuclease	$8x10^{-10}$ - $4x10^{-9}$	2/5	0/5	0/5	0/5	2/20	
Q99XV9_STRP1	*dppA, M5005_Spy1704, SPy_2000*	Dipeptide-binding protein	$8x10^{-7}$ - $1x10^{-6}$	2/5	0/5	0/5	0/5	2/20	
Q99YA0_STRP1	*M5005_Spy1530, SPy_1798*	Putative Fe3+-siderophore transport protein	$2x10^{-12}$ - $2x10^{-10}$	2/5	0/5	0/5	0/5	2/20	
Q99YA2_STRP1	*M5005_Spy1528, SPy_1795*	Ferrichrome-binding protein	$6x10^{-12}$ - $8x10^{-8}$	1/5	1/5	0/5	0/5	2/20	<25%
Q99ZN4_STRP1	*M5005_Spy0875, SPy_1154*	Sortase	$8x10^{-8}$ - $1x10^{-6}$	1/5	0/5	0/5	1/5	2/20	
G6PI_STRP1	*pgi, SPy_0215, M5005_Spy0185*	Glucose-6-phosphate isomerase	$6x10^{-8}$	0/5	0/5	0/5	1/5	1/20	
Q48WD9_STRP1	*sic1.01, M5005_Spy1718*	Streptococcal inhibitor of complement	$1x10^{-30}$	1/5	0/5	0/5	0/5	1/20	
Q48X28_STRP1	*manL, M5005_Spy1479*	PTS system, mannose-specific IIAB component	$6x10^{-11}$	1/5	0/5	0/5	0/5	1/20	
Q7DAL3_STRP1	*scl, sclA, M5005_Spy1687, SPy_1983*	Collagen-like surface protein	$1x10^{-7}$	1/5	0/5	0/5	0/5	1/20	
Q7DAM2_STRP1	*mac, M5005_Spy0668, SPy_0861*	IgG-degrading protease	$7x10^{-11}$	1/5	0/5	0/5	0/5	1/20	
Q99Z26_STRP1	*mf3, SPy_1436*	Putative deoxyribonuclease	$4x10^{-10}$	1/5	0/5	0/5	0/5	1/20	
Q99Z67_STRP1	*gapN, M5005_Spy1119, SPy_1371*	NADP-dependent glyceraldehyde-3-phosphate dehydrogenase	$3x10^{-8}$	1/5	0/5	0/5	0/5	1/20	
Q99ZU1_STRP1	*M5005_Spy0793, SPy_1070*	Putative dipeptidase	$2x10^{-9}$	0/5	0/5	0/5	1/5	1/20	
Q99ZX5_STRP1	*acoL, M5005_Spy0755, SPy_1031*	Dihydrolipoamide dehydrogenase	$6x10^{-4}$	1/5	0/5	0/5	0/5	1/20	
Q9A1E5_STRP1	*M5005_Spy0270, SPy_0317*	ABC transporter substrate-binding protein	$9x10^{-7}$	0/5	0/5	1/5	0/5	1/20	
Q9A068_STRP1	*bcaT, M5005_Spy0713, SPy_0911*	Branched-chain-amino-acid aminotransferase	$6x10^{-6}$	1/5	0/5	0/5	0/5	1/20	
Q9A146_STRP1	*SPy_0470*	67 kDa Myosin-crossreactive streptococcal antigen	$9x10^{-6}$	1/5	0/5	0/5	0/5	1/20	
Q9A1E4_STRP1	*M5005_Spy0271, SPy_0319*	ABC transporter substrate-binding protein	$1x10^{-6}$	0/5	0/5	0/5	1/5	1/20	
RPOA_STRP1	*rpoA, SPy_0080, M5005_Spy0070*	DNA-directed RNA polymerase subunit alpha	$6x10^{-6}$	1/5	0/5	0/5	0/5	1/20	
SYA_STRP1	*alaS, SPy_1389, M5005_Spy1132*	Alanyl-tRNA synthetase	$1x10^{-5}$	1/5	0/5	0/5	0/5	1/20	
SYS_STRP1	*serS, SPy_1742, M5005_Spy1483*	Seryl-tRNA synthetase	$2x10^{-10}$	1/5	0/5	0/5	0/5	1/20	
Y652_STRP1	*SPy_0652, M5005_Spy0539*	UPF0042 nucleotide-binding protein	$9x10^{-10}$	1/5	0/5	0/5	0/5	1/20	

Supplementary Table 3: The *S. pyogenes* antigens purified by comparative immunoprecipitation. Numbers represent the proportion of strains from which targets were identified. Targets identified from ≥75% of strains using one or more E-IVIG preparations are displayed.

		Endobulin					Intratect					Privigen				
**Uniprot Identifier**	**Protein product**	**M1**	**M12**	**M3**	**M89**	**Total**	**M1**	**M12**	**M3**	**M89**	**Total**	**M1**	**M12**	**M3**	**M89**	**Total**
C5AP_STRP1	C5a peptidase	2/2	2/2	2/2	2/2	8/8	2/2	2/2	2/2	2/2	8/8	2/2	2/2	2/2	2/2	8/8
DNAK_STRP1	Chaperone protein DnaK	2/2	2/2	2/2	2/2	8/8	2/2	2/2	2/2	2/2	8/8	2/2	2/2	2/2	2/2	8/8
EFTS_STRP1	Elongation factor Ts	2/2	2/2	2/2	2/2	8/8	2/2	2/2	2/2	2/2	8/8	2/2	2/2	2/2	2/2	8/8
MTSA_STRP1	Metal ABC transporter substrate-binding lipoprotein	2/2	2/2	2/2	2/2	8/8	2/2	2/2	2/2	2/2	8/8	2/2	2/2	2/2	2/2	8/8
PGK_STRP1	Phosphoglycerate kinase	2/2	2/2	2/2	2/2	8/8	2/2	2/2	2/2	2/2	8/8	2/2	2/2	2/2	2/2	8/8
Q48Y96_STRP1	Maltose/maltodextrin-binding protein	2/2	2/2	2/2	2/2	8/8	2/2	2/2	2/2	2/2	8/8	2/2	2/2	2/2	2/2	8/8
Q490V0_STRP1	Oligopeptide-binding protein	2/2	2/2	2/2	2/2	8/8	2/2	2/2	2/2	2/2	8/8	2/2	2/2	2/2	2/2	8/8
Q491G2_STRP1	Nucleoside-binding protein	2/2	2/2	2/2	2/2	8/8	2/2	2/2	2/2	2/2	8/8	2/2	2/2	2/2	2/2	8/8
Q7DAN2_STRP1	Nicotine adenine dinucleotide glycohydrolase	2/2	2/2	2/2	2/2	8/8	2/2	2/2	2/2	2/2	8/8	2/2	2/2	2/2	2/2	8/8
Q99XX8_STRP1	Putative pullulanase	2/2	2/2	2/2	2/2	8/8	2/2	2/2	2/2	2/2	8/8	2/2	2/2	2/2	2/2	8/8
Q99ZH4_STRP1	Nucleoside-binding protein	2/2	2/2	2/2	2/2	8/8	2/2	2/2	2/2	2/2	8/8	2/2	2/2	2/2	2/2	8/8
Q99ZW9_STRP1	Hypothetical membrane associated protein	2/2	2/2	2/2	2/2	8/8	2/2	2/2	2/2	2/2	8/8	2/2	2/2	2/2	2/2	8/8
Q9A0C0_STRP1	Cell surface protein	2/2	2/2	2/2	2/2	8/8	2/2	2/2	2/2	2/2	8/8	2/2	2/2	2/2	2/2	8/8
Q9A0J7_STRP1	Endonuclease/exonuclease/phosphatase family protein	2/2	2/2	2/2	2/2	8/8	2/2	2/2	2/2	2/2	8/8	2/2	2/2	2/2	2/2	8/8
Q9A1G0_STRP1	Penicillin-binding protein	2/2	2/2	2/2	2/2	8/8	2/2	2/2	2/2	2/2	8/8	2/2	2/2	2/2	2/2	8/8
Q9A1H3_STRP1	SpyAD	2/2	2/2	2/2	2/2	8/8	2/2	2/2	2/2	2/2	8/8	2/2	2/2	2/2	2/2	8/8
Q9A1Z8_STRP1	Putative secreted protein	2/2	2/2	2/2	2/2	8/8	2/2	2/2	2/2	2/2	8/8	2/2	2/2	2/2	2/2	8/8
TACY_STRP1	Streptolysin O	2/2	2/2	2/2	2/2	8/8	2/2	2/2	2/2	2/2	8/8	2/2	2/2	2/2	2/2	8/8

Supplementary Table 3 continued:

		Endobulin					Intratect					Privigen				
**Uniprot Identifier**	**Protein product**	**M1**	**M12**	**M3**	**M89**	**Total**	**M1**	**M12**	**M3**	**M89**	**Total**	**M1**	**M12**	**M3**	**M89**	**Total**
G3P_STRP1	Glyceraldehyde-3-phosphate dehydrogenase	1/2	2/2	2/2	2/2	7/8	1/2	2/2	2/2	2/2	7/8	1/2	2/2	2/2	2/2	7/8
PILIN_STRP1	Pilin	2/2	2/2	2/2	1/2	7/8	2/2	2/2	2/2	1/2	7/8	2/2	2/2	2/2	1/2	7/8
Q490K8_STRP1	SpyCEP	1/2	2/2	2/2	2/2	7/8	1/2	2/2	2/2	2/2	7/8	1/2	2/2	2/2	2/2	7/8
Q99XV0_STRP1	M protein type 1	2/2	2/2	2/2	1/2	7/8	2/2	2/2	2/2	1/2	7/8	2/2	2/2	2/2	1/2	7/8
Q99ZT1_STRP1	Polysaccharide deacetylase	2/2	2/2	1/2	2/2	7/8	2/2	2/2	1/2	2/2	7/8	2/2	2/2	1/2	2/2	7/8
Q9A156_STRP1	Putative cyclophilin-type protein	1/2	2/2	2/2	2/2	7/8	1/2	1/2	2/2	1/2	5/8	2/2	2/2	1/2	2/2	7/8
PEPDB_STRP1	Probable dipeptidase B	2/2	1/2	1/2	2/2	6/8	2/2	1/2	2/2	1/2	6/8	2/2	1/2	1/2	2/2	6/8
Q99XV4_STRP1	Streptococcal histidine triad protein	2/2	1/2	1/2	2/2	6/8	2/2	1/2	0/2	2/2	5/8	2/2	1/2	1/2	1/2	5/8
Q99YL6_STRP1	Putative uncharacterized protein	1/2	1/2	1/2	2/2	5/8	2/2	1/2	0/2	2/2	5/8	1/2	2/2	1/2	2/2	6/8
Q9A0C5_STRP1	Putative uncharacterized protein	2/2	0/2	1/2	2/2	5/8	2/2	0/2	1/2	1/2	4/8	2/2	2/2	1/2	2/2	7/8
Q99XU2_STRP1	Periplasmic component of efflux system	1/2	1/2	0/2	2/2	4/8	0/2	1/2	0/2	2/2	3/8	2/2	1/2	2/2	2/2	7/8
Q9A147_STRP1	Putative 42 kDa protein	0/2	1/2	2/2	0/2	3/8	2/2	2/2	2/2	1/2	7/8	0/2	2/2	2/2	0/2	4/8
